# A risk score to predict 30-day hospital readmission rate in cirrhotic patients with spontaneous bacterial peritonitis

**DOI:** 10.1186/s40001-023-01126-2

**Published:** 2023-05-12

**Authors:** Nasser Mousa, Ahmed Abdel-Razik, Sherif Elbaz, Mohamed Salah, Mohammed Abdelaziz, Alaa Habib, Ahmed Deib, Abdel-Naser Gadallah, Niveen El-wakeel, Waleed Eldars, Narmin effat, Ola El-Emam, Khaled Taha, Alaa Elmetwalli, Eman Mousa, Dina Elhammady

**Affiliations:** 1 Tropical Medicine Department, Mansoura University, Mansoura, Egypt; 2 Damietta Cardiology and Gastroenterology Center, Damietta, Egypt; 3 Endemic Diseases and Gastroenterology Department, Aswan University, Aswan, Egypt; 4 Internal Medicine Department, Mansoura University, Mansoura, Egypt; 5 Internal Medicine Department, Menuofia University, Menuofia, Egypt; 6 Medical Microbiology and Immunology Department, Mansoura University, Mansoura, Egypt; 7 Department of Basic Medical Sciences, Faculty of Medicine, New Mansoura University, Mansoura, Egypt; 8 Clinical Pathology Department, Mansoura University, Mansoura, Egypt; 9 Department of Clinical Trial Research Unit and Drug Discovery, Egyptian Liver Research Institute and Hospital (ELRIAH), Mansoura, Egypt; 10 Faculty of Dentistry, Mansoura University, Mansoura, Egypt

**Keywords:** Spontaneous bacterial peritonitis, 30-day readmission rate, Liver cirrhosis

## Abstract

**Background and aim:**

There is lack of 30-day hospital readmission prediction score in patients with liver cirrhosis and SBP. The aim of this study is to recognize factors capable of predicting 30-day readmission and to develop a readmission risk score in patients with SBP.

**Methods:**

This study prospectively examined the 30-day hospital readmission for patients previously discharged with a diagnosis of SBP. Based on index hospitalization variables, a multivariable logistic regression model was implemented to recognize predictors of patient hospital readmission within 30 days. Consequently, Mousa readmission risk score was established to predict 30-day hospital readmission.

**Results:**

Of 475 patients hospitalized with SBP, 400 patients were included in this study. The 30-day readmission rate was 26.5%, with 16.03% of patients readmitted with SBP. Age ≥ 60, MELD > 15, serum bilirubin > 1.5 mg/dL, creatinine > 1.2 mg/dL, INR > 1.4, albumin < 2.5 g/dL, platelets count ≤ 74 (10^3^/dL) were found to be independent predictors of 30-day readmission. Incorporating these predictors, Mousa readmission score was established to predict 30-day patient readmissions. ROC curve analysis demonstrated that at a cutoff value ≥ 4, Mousa score had optimum discriminative power for predicting the readmission in SBP with sensitivity 90.6% and specificity 92.9%. However, at cutoff value ≥ 6 the sensitivity and specificity were 77.4% and 99.7%, respectively, while a cutoff value ≥ 2 had sensitivity of 99.1% and specificity of 31.6%.

**Conclusions:**

The 30-day readmission rate of SBP was 25.6%. With the suggested simple risk assessment Mousa score, patients at high risk for early readmission can be easily identified so as to possibly prevent poorer outcomes.

**Supplementary Information:**

The online version contains supplementary material available at 10.1186/s40001-023-01126-2.

## Introduction

Patients with liver cirrhosis who develop ascites generally have a poor prognosis marked by high morbidity and mortality [1]. The development of ascites in cases of cirrhosis is multifactorial in origin, with portal hypertension accounting for about 75% of cases and a variety of inflammatory, infectious, and malignant conditions making up the remaining cases [2–6]. Ascitic fluid often becomes infected with bacteria in absence of any apparent intra-abdominal source of infection or malignant infiltration in a condition called spontaneous bacterial peritonitis (SBP) [7], which occurs in cirrhotic patients with ascites at a rate of about 10–25%. Affliction with SBP detrimentally affects the prognosis of these patients, resulting in increased liver decompensation, associated sepsis, and ultimately, multi-organ failure [8–10]. Furthermore, SBP patients have high mortality rates at 20–40% [11, 12] with a 1-year recurrence rate of 40–70% [13]. These rates of mortality and recurrence are expected to increase with the emergence of multi-drug resistant bacteria as the causative agent responsible for SBP [14].

A measure of hospital quality and performance is the rate of patient 30-day hospital readmissions, which consequently have a massive effect on the overall cost of health care. This is determined by the annual post-index hospitalization charges for patients with a 30-day readmission which were considerably higher than for patients admitted after 30 days or not readmitted at all [15].

The healthcare system remains tasked with early hospital readmission for patients with cirrhosis. While classification of risk may aid in allocating resources, the available current models [16–21], summarized in Additional file 2: Table S1 by Koola et al. [22], were limited by use of small sample size, single-institution cohorts or had modest performance. In addition, most of these reports primarily aimed to detect risk factors rather than building a risk prediction model [23]. In addition, only two of the studies attempted to assess calibration. Their method of the calibration was the Hosmer–Lemeshow test, which has been criticized for its incompleteness and inadequacy as an evaluation of calibration. Illustratively, the Hosmer–Lemeshow test only measures calibration at a few points, and does not adequately evaluate the overall accuracy of the model's predictions. In addition, the test does not assess discrimination, which is the ability of the model to correctly identify those with and without the outcome being studied.

It is well-recognized that readmission of SBP is associated with worse clinical course as well as greater medical, economic, and psychosocial load on patients [24].

Because of the massive lack of data on the characteristics of 30-day readmission, as well as the shortage of accurate readmission rates and predictors for 30-day readmissions in patients with SBP, this study aimed to compare hospitalization characteristics for index and 30-day readmission cases of SBP and to generate a novel score to predict 30-day readmissions in patients with spontaneous bacterial peritonitis complicating liver cirrhosis (Additional file 1: Figure S1).

## Patients and methods

This prospective cohort study included 475 cases, aged ≥ 18 years, with a primary discharge diagnosis of spontaneous bacterial peritonitis. Patients were recruited from the Tropical and Internal Medicine Departments, Mansoura University, Department of Tropical Medicine, Menoufia University, and Damietta Cardiology and Gastroenterology Center from October 2020 to Jun 2022. Re-hospitalization within 30 days from index admission was documented. All cases were subjected to complete history taking followed by clinical and radiological examination with abdominal ultrasonography and triphasic CT as indicated. Laboratory analysis, diagnostic paracentesis, and analysis of ascitic fluid were performed for all patients.

Exclusion criteria included non-cirrhotic ascites, e.g., tuberculous ascites, patients with heart or renal failure, malignancy, sepsis, secondary bacterial peritonitis, as well as patients with unrelated treated infection. Also excluded were patients who died during follow-up period or were missing information on readmission. All discharged patients were maintained on prophylactic antibiotics.

SBP was diagnosed based on the common practice guidelines of ascitic fluid polymorphonuclear neutrophil cell count being ≥ 250/m without evidence of any other cause of peritonitis or hemorrhagic ascites [25, 26]. When indicated, ascitic fluid was further investigated with Ziehl–Neelsen staining [27]. At the time of ascitic fluid collection, blood samples were also obtained. The 5 mL of venous blood collected were divided as 1 mL into a tube containing EDTA for CBC, while 4 mL were added to another tube and left to clot. Centrifugation separated the non-hemolyzed sera which was used to assess status of liver functions, including bilirubin, liver enzymes ALT and AST, albumin, and prothrombin time, as well as determination of blood sugar, creatinine, and urea. Tumor markers alpha-fetoprotein and carcinoembryonic antigen were also quantified.

After improvement of ascitic fluid count and clinical condition, discharged patients included in this study were followed up every week for 30 days, either by outpatient visit or telemedicine, for any change or deterioration in clinical state and readmission when indicated.

This study was approved by Mansoura Faculty of Medicine Institutional Review Board “MFM-IRB”. All patients were provided written informed consent prior to participation in any protocol-specific procedure.

### Statistical analysis

Statistical analyses were performed via SPSS Corp (IBM Corp). Number and percent were used to describe qualitative data while, after testing normality using Kolmogrov–Smirnov test, quantitative data required use of median (minimum and maximum) for non-parametric variables and mean ± SD for parametric data. Comparing between two groups was done with either *t* test for parametric variables or Mann–Whitney test for non-parametric data, with comparison between two or more groups required use of χ^2^ test or Monte Carlo tests.

Receiver operator characteristics (ROC) curve allowed choosing the cutoff point with the highest sensitivity and specificity rates while stepwise logistic regression was used for multivariable regression. In addition, adjusted Odds ratio with 95% confidence interval were calculated and linear regression analysis used for prediction of independent variables of continuous parametric outcome. Kaplan–Meier curve was used to demonstrate time to event. A *p* value < 0.05 was considered statistically significant.

## Results

### Readmission rates and common admitting diagnosis

**Fig. 1:**
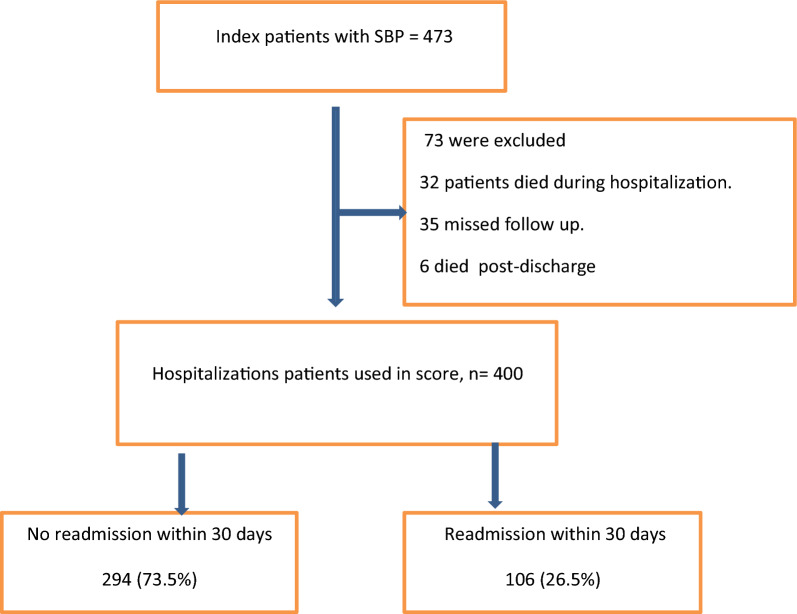
Follow chart of study patients

**Fig. 2:**
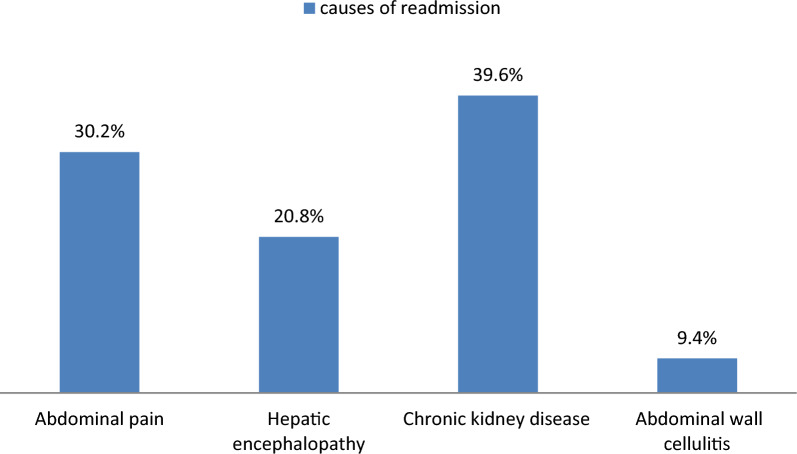
Causes of readmission

At index admission, a total of 473 patients with SBP were included in this study. Seventy-three patients were excluded (32 patients died during index hospitalization, 35 patients missed follow-up checks, and 6 died following discharge). Of the included patients, 400 patients who met the eligibility criteria were included in the study. One hundred and six patients (26.5%) were readmitted within 30 days. SBP was identified as the readmitting diagnosis in 17/106 patients (16.03%) of these 30-day readmissions (Fig. 1). Chronic kidney disease was the most common presentation on readmission (39.6%), followed by abdominal pain (30.2%), hepatic encephalopathy (20.8%), and abdominal wall cellulitis (9.4%) (Fig. 2).

**Fig. 3:**
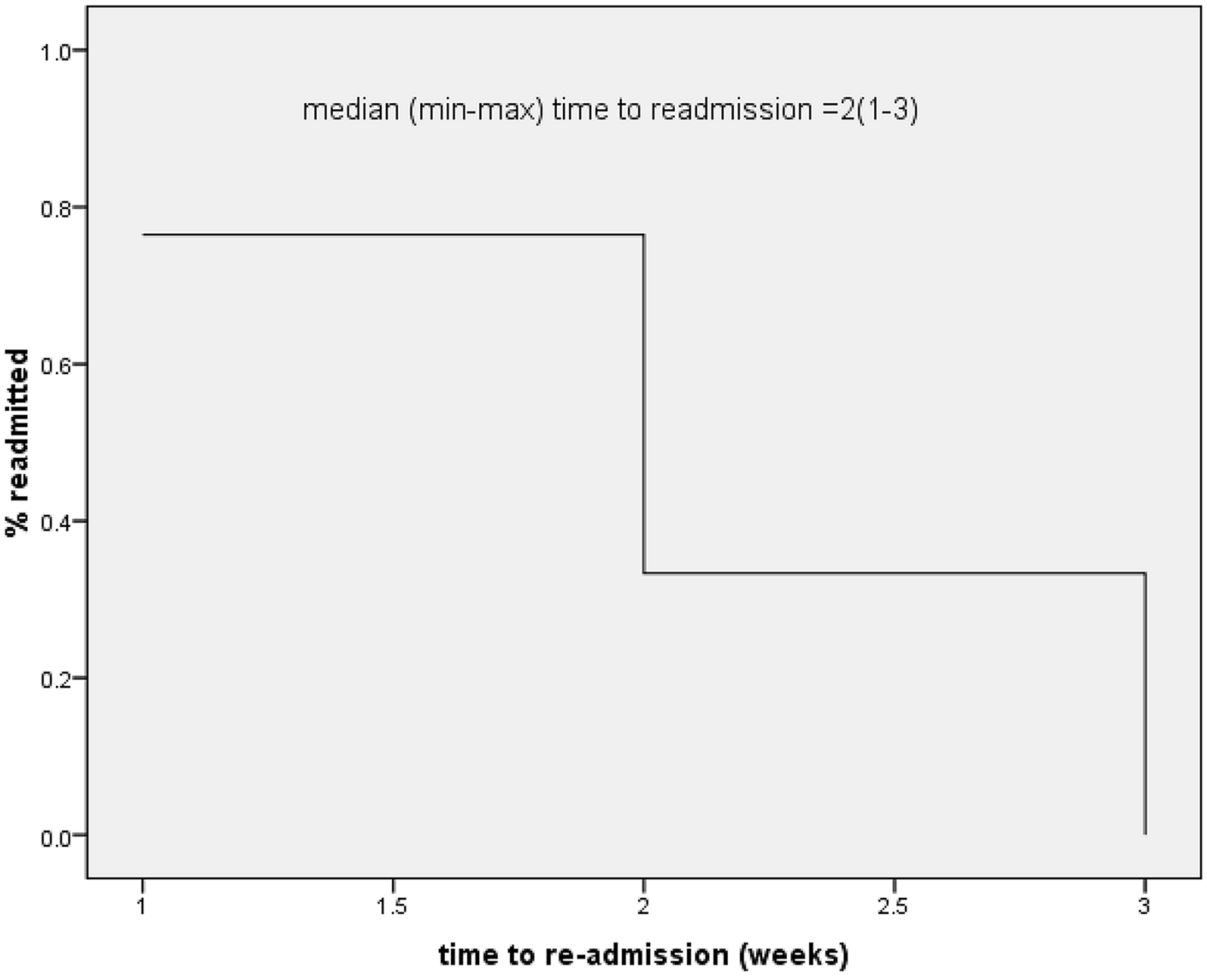
Time of readmission

Figure 3 shows that the majority of patients (41.4%) were readmitted within 2 weeks of their discharge, 22.6% were readmitted within 1 week, 34% were readmitted within third week and no readmission occurred in fourth week**.**

Table 1 shows univariate analysis of patients who had readmission and patients without readmission within 30-day post-discharge. A statistically significant association was found between higher incidence of readmission and age ≥ 60 years (39.1% of cases aged ≥ 60 years vs 19.8% of cases aged < 60 years, *p* = 0.001; OR = 2.59, 95%CI 1.64–4.1), presence of chronic kidney disease (66.7% vs 33.3%, *p* = 0.001; OR = 5.91, 95%CI 1.74–20.08), hepatic encephalopathy (29.2% vs 15.4%, *p* = 0.013; OR = 0.441, 95%CI 0.228–0.854), Child score (29.06% child C vs 16.25% Child B, *p* = 0.020; OR = 2.11, 95%CI 1.11–4.01) and MELD 15 (53.9% vs 46.1%, *p* = 0.001; OR = 8.93, 95%CI 5.39–14.78). Furthermore, decreasing hospital readmission was found with ceftriaxone therapy (34.2% vs 65.8%, *p* = 007; OR = 1.84, 95%CI 1.17–2.89) and levofloxacin treatment of SBP (6.2% vs 93.8%, *p* = 006; OR = 0.169, 95%CI 0.039–0.721). However, no significant difference was found regarding other compared data.

**Table 1:** Univariate analysis of factors present on first admission associated with readmission

Variable	Total *N* = 400	Readmissio*n* (*N* = 106) (*N*/%)	No readmission (*N* = 294) (*N*/%)	*p* value	COR (95%CI)
*Age (years)* < 60 ≥ 60	262 138	52 (19.8) 54 (39.1)	210 (80.2) 84 (60.9)	< 0.001*	R 2.59(1.64–4.10)
*Sex* Male Female	262 138	74 (28.2) 32 (23.2)	188 (71.8) 106 (76.8)	0.276	1.30(0.808–2.10) R
*Comorbidities* Non Diabetes mellitus Hypertension CKD	268 96 24 12	68 (25.4) 26 (27.1) 4 (16.7) 8 (66.7)	200 (74.6) 70 (72.9) 20 (83.3) 4 (33.3)	0.466 0.881 0.260 0.001	0.841(0.528–1.34) 1.04 (0.619–1.74) 0.537(0.179–1.60) 5.91(1.74–20.08)
*Fever* Yes No	6 394	2 (33.3) 104 (26.4)	4 (66.7) 290 (73.6)	0.657	1.39 (0.252–7.72) R
*Abdominal pain* Yes No	206 194	60(29.1) 46(23.7)	146 (70.9) 148 (76.3)	0.22	1.32 (0.845–2.07) R
*AWC* Yes No	54 346	18(33.3) 88(25.4)	36(66.7) 258(74.6)	0.221	1.47(0.792–2.71) R
*Oesophageal varices* Yes No	288 112	84 (29.2%) 22 (19.6%)	204 (70.8%) 90 (80.4%)	0.052	1.68 (0.79–3.56) 1r
*HE* No Yes	78 322	12 (15.4%) 94 (29.2%)	66 ( 70.8) 228 (84.6)	0.013	0.441(0.228–0.854) R
*HCC* Yes No	88 312	16 (18.2%) 90 (28.7%)	70 (81.4%) 224 (71.3%)	0.61	1r 1.76 (0.969–3.18)
*Child score* Child B Child C	80 320	13 (16.25%) 93 (29.06%)	67 (83.75%) 227 (70.93%)	0.020	2.11(1.11–4.01) R
*MELD score (N/%)* ≤ 15 > 15	259 141	30 (11.6) 76 (53.9)	229 (88.4) 65 (46.1)	< 0.001	R 8.93(5.39–14.78)
Antibiotic taken Cefotaxime Ceftriaxone Levofloxacin Impienem Cefoprazone	210 146 32 8 4	106 52 (24.8) 50 (34.2) 2 (6.2) 2 (25) 0	294 158 (75.2) 96 (65.8) 30 (93.8) 6 (75) 4 (100)	0.407 0.007 0.006 0.922 0.227	0.829(0.532–1.29) 1.84(1.17–2.89) 0.169(0.039–0.721) 0.923(0.184–4.65) Undefined

CKD, chronic kidney disease; AWC, abdominal wall cellulitis; HE, hepatic encephalopathy; HCC, hepatocellular carcinoma

Table 2 shows laboratory data of readmitted patients in comparison with non-readmitted patients at index admission. Compared to patients without readmission, patients with 30-day readmission had low serum albumin and platelets count, and increased serum bilirubin, INR, and serum creatinine. However, no significant change was detected with regard to ascitic fluid polymorphonuclear neutrophil, haemoglobin, WBCs, ALT, and AST.

**Table 2:** Comparison between laboratory data in readmission and no readmission

Parameters	Re-admission (*n* = 106)	No readmission (*n* = 294)	*p* value
Ascitic polymorph nuclear neutrophil (cells/mm^3^) Median (Min–Max)	400 (250–2700)	350 (300–1800)	0.169
HB (g/dL) Mean ± SD	9.33 ± 1.26	9.32 ± 1.87	0.171
WBCs (cells/mm^3^) Median (Min–Max)	5.3(1.8–13)	5(1.4–23)	0.980
PLT (cells/mm^3^) Median (Min–Max)	69)15–163)	75 (25–500)	0.041
Albumin (g/dL) Mean ± SD	2.13 ± 0.53	2.41 ± 0.56	< 0.001
Bilirubin (mg/dL): Median (Min–Max)	1.6 (0.6–23)	1(0.15–6.8)	< 0.001
ALT (U/L): Median (Min–Max)	41(18–84)	37(14–255)	0.351
AST (U/L): Median (Min–Max)	44 (20–105)	44(18–869)	0.545
INR: Mean ± SD	1.6 ± 0.40	1.46 ± 0.39	< 0.001
Creatinine (mg/dL): Median (Min–Max)	1.8 (0.7–7)	1.0 (0.2–2.5)	0.001

HB, hemoglobin; PLT, platelets; ALT, alanine aminotransferase; AST, aspartate aminotransferase

Table 3 shows multivariate analysis of factors associated with the increased readmission rate. There was a statistically significant association between readmission and age ≥ 60 (*p* = 0.00, AOR: 2.59, 95% CI 1.64–4.10), MELD > 15 (*p* = 0.001; AOR: 2.43. 95% CI 1.48–4.02) and serum albumin < 2.5 g/dL (*p* = 0.003, AOR: 2.06, 95% CI 1.27–3.34), platelets count ≤ 74 (10^3^/dL) (*p* = 0.025, AOR: 1.67, 95% CI 1.07–2.63), serum bilirubin 1.5 mg/dL (*p* < 0.00, AOR: 3.21, 95% CI 1.95–5.27), serum creatinine > 1.2 mg/dL (*p* < 0.001, AOR: 3.37, 95% CI 2.08–5.45) and INR > 1.4 (*p* < 0.001, AOR: 2.48, 95% CI 1.55–4.0).

**Table 3:** Multivariate analysis of factors associated with re-admission

Parameter	β	P value	AOR (95%CI)
*Age (years)* < 60(r) ≥ 60	0.954	0.001	2.59 (1.64–4.10)
*MELD score* ≤ 15 (r) > 15	0.892	0.001	2.43 (1.48–4.02)
*Child score* Child B Child C	1.25	0.09	1.25 (0.65–6.8)
*Albumin* < 2.5 ≥ 2.5 (r)	0.725	0.003	2.06 (1.27–3.34)
*Platelets (cells/mm*^*3*^*)* ≤ 74 > 74 (r)	0.517	0.025	1.67 (1.07–2.63)
*Bilirubin (mg/dL)* ≤ 1.5 (r) > 1.5	1.165	< 0.001	3.21(1.95–5.27)
*Creatinine* ≤ 1.2 (r) > 1.2	1.21	< 0.001	3.37 (2.08–5.45)
*INR* ≤ 1.4 (r) > 1.4	0.912	< 0.001	2.48 (1.55–4.0)
Chronic kidney disease	0.154	0.07	1.25 (1.14–6.51)
Hepatic encephalopathy	– 0.125	0.07	0.412 (0.311–1.25)
Constant % correctly predicted Model *χ*^2^	– 1.98 73.5% 29.34, *p* < 0.001*		

*β*: regression coefficient, AOR: Adjusted Odds Ratio, CI: Confidence Interval. P value ≤ 0.05 is considered statistically significant

## 30 DAY Mousa readmission risk model

Based on the predictors associated with 30-day Mousa readmission risk, a novel scoring system was generated to determine the risk of readmission. Age ≥ 60 years, MELD > 15, serum albumin < 2.5 g/dL, serum bilirubin > 1.5 mg/dL, platelets count ≤ 7 4 (10^3^/dL), serum creatinine > 1.2 mg/dL and INR > 1.4 were identified as predictors for 30-day readmission risk. In the score proposed by the this study group, one point was allocated for each predictor of risk with 0 points allocated in no predictive factor is present (Table 4, Additional fig. 1).

**Table 4:** Mousa scoring system for prediction of readmission in spontaneous bacterial peritonitis

Parameter	Β coefficient	Score
*Age (years)*		
< 60	**0**	**0**
≥ 60	0.954	**1**
*MELD score*		
≤ 15	0	0
> 15	0.892	1
*Albumin*		
Albumin ≥ 2.5	0	**0**
Albumin < 2.5	0.725	**1**
*Bilirubin (mg/dL)*		
≤ 1.5	0	**0**
> 1.5	1.165	**1**
*Platelets (cells/mm*^*3*^*)*		
≤ 74	0.517	**1**
> 74	0	**0**
*Creatinine*		
≤ 1.2(r)	0	**0**
> 1.2	1.21	**1**
*INR*		
≤ 1.4(r)	0	**0**
> 1.4	0.912	**1**

**Fig. 4:**
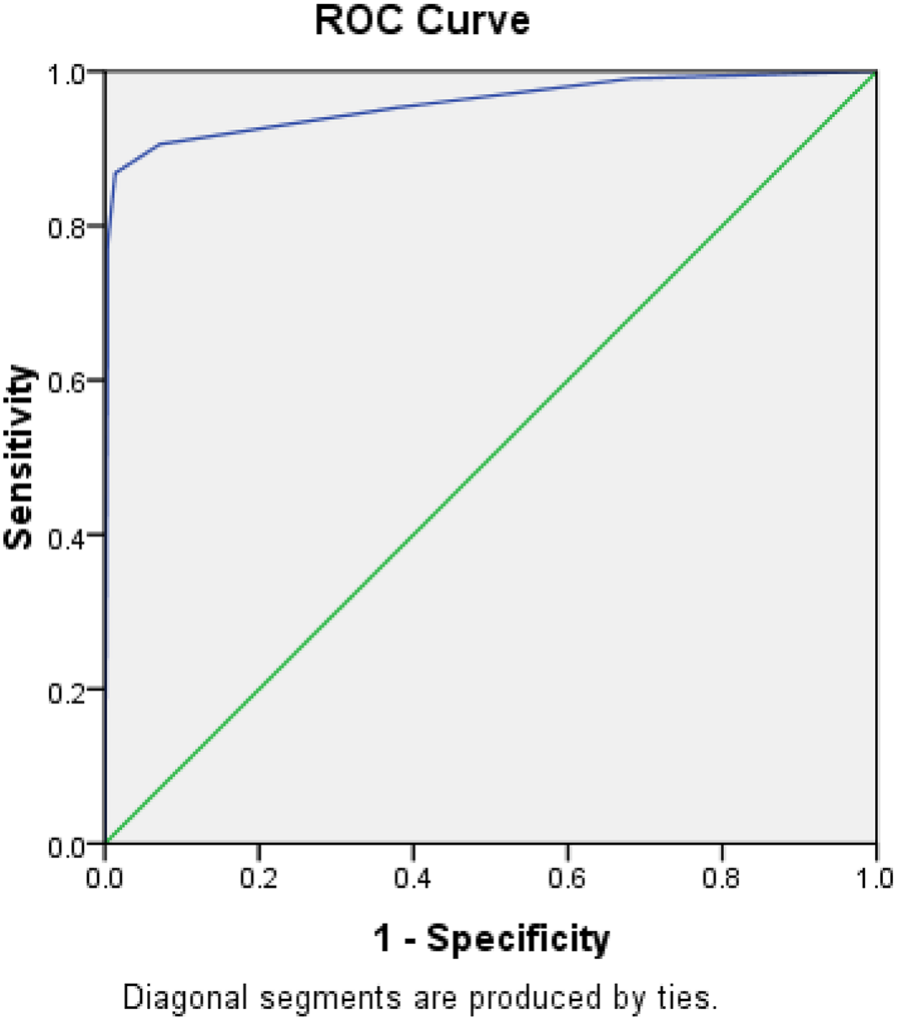
ROC curve analysis of calculated score at different cut off values

Table 5 shows ROC curve analysis of calculated score at different cut off values. The ROC curve analysis (Fig. 4) demonstrated that at a cutoff value ≥ 4, Mousa score had optimum discriminative power for predicting readmission in cirrhotic SBP patients with sensitivity of 90.6% and specificity of 92.9%. However, at a cutoff value ≥ 6, the sensitivity and specificity were 77.4% and 99.7%, respectively, while at cutoff value ≥ 2 the sensitivity was 99.1% and specificity was 31.6%.

**Table 5:** Roc curve analysis of Mousa score for detection of hospital readmission in patients with spontaneous bacterial peritonitis

	AUC (95%CI)	Cut off point	P value	Sensitivity %	Specificity%
Scores	0.958 (0.931–0.984)	≥ 2	< 0.001	99.1	31.6
		≥ 3		95.3	62.2
		≥ 4		90.6	92.9
		≥ 5		86.8	98.6
		≥ 6		77.4	99.7

## Discussion

The issue of readmission in cirrhotic SBP patients is currently at an acute crossroads [28]. It is well-known that bacterial infections cause significant morbidity and mortality in patients in cirrhosis [29, 30]. While the 30-day period for SBP patient readmission selected by the Centers for Medicare and Medicaid Services is considered a measure of quality clinical performance and economic consequences [31], the 30-day hospital readmission rate is also considered the main indicator of quality and aim for charge reduction [32].

Because SBP is the most common infection in seen in cirrhotic patients [33, 34], the American Association for the Study of Liver Diseases guidelines recommend prophylactic antibiotic therapy in recovered cirrhotic SBP patients to prevent recurrence of this infection [35]. However, the prevalence of multi-drug resistant organisms has boosted the recurrence rate to nearly 70% [36, 37], with some reports showing recurrence rates in norfloxacin-receiving patients as high as in initial studies [38].

While studies have repeatedly shown that readmission of cirrhotic patients is a common occurrence [16–22], a risk score to predict 30-day hospital readmissions rate of cirrhotic spontaneous bacterial peritonitis patients has not been designed. In the current study, patients discharged with a primary diagnosis of spontaneous bacterial had a 30-day hospital readmission rate of 26.5%. This is in accordance with a recent study by Dahiya et al. who found that there was a 30% readmission rate of SBP at 30 days in the USA [24]. In our study, independent predictors for these readmissions were found to be age ≥ 60 Y, MELD > 15, serum albumin < 2.5 g/dL, platelets count ≤ 74 (10^3^/cmm), serum bilirubin > 1.5 mg/dL, serum creatinine > 1.2 mg/dL, and INR > 1.4.

It is common knowledge that in clinical practice, formulation of a good risk score is based on certain patient- and disease-specific characteristics identified during index hospitalization. Therefore, using the administration database, predictors of hospital readmission were assessed and proposed in a simple applicable risk model, coined the Mousa scoring system, which utilized factors identified at index hospital admission and during required inpatient stay of cirrhotic SBP patients so as to assess the risk of 30-day readmission. To our knowledge, this is the first report to propose a model for risk readmission in SBP patients.

Moussa score allocates one point for each risk predictor if present and 0 points if not present for a total sum of 7 points. Interestingly, it was found that the score had a specificity for prediction of hospital readmission that increased with upgrading of the score from 31.6% when the score was ≥ 2 points to a maximum specificity of 99.7% when the score was ≥ 6 points. Conversely, the sensitivity decreased with downgrading of the score from maximum sensitivity of 99.1% when the score was ≥ 2 points to reach 77.4% when the score was ≥ 6 points. However, when the score was ≥ 4 points the scoring system had excellent sensitivity and specificity for prediction of hospital readmission in patients with SBP at 90.6% and 92.9, respectively, with AUC of 0.958.

Previous papers reported an increased incidence of SBP in the elderly [34], possibly due to worsening of liver state and additional comorbidity in these patients which increased susceptibility to infectious [39]. In the current study, age of more than 60 years was associated with higher readmission rate, therefore, allocating age ≥ 60 years with one point. Similarly, Chirapongsathorn et al. [40] found that age over 65 years was a main risk factors for index hospitalization. In addition, in his model for prediction of 30-day hospital readmission risk in cirrhotic patients, Koola et al. also depended on age of 60 years [22]. This may explain the increased readmission rate in older patients in addition to poor follow-up following hospital discharge in this group of patients.

The severity of liver disease during index hospitalization in the present study was represented both by Model for End-Stage Liver Disease (MELD) and Child–Pugh–Turcotte (CPT) scores at index admission. Both scores were significant in patients with readmission compared to non-readmitted patients in univariate analysis. However, using multivariate analysis to determine a predictor associated with readmissions, MELD score was the only significant score and, therefore, was included in the risky Mousa score. This perhaps add credibility to Mousa score, because the excluded Child score included 2 subjective variables, namely, ascites and hepatic encephalopathy, that may be subject to observer judgement and to therapy with diuretics and lactulose [41]. Earlier studies had similarly demonstrated that MELD score was linked to increased risk of readmission in cirrhotic patients [17–19]. Conversely, Koola et al. found that MELD score had low superiority in his mortality risk prediction model for patients with cirrhosis when compared to his model [42]. Furthermore, Mousa score incorporated the three non-subjective components (albumin, bilirubin, and INR) of Child–Pugh–Turcotte (CPT) score.

This study also found thrombocytopenia to be a predictor for SBP-related readmission. In his prediction models for hospital readmission for patients with cirrhosis, Berman et al. found that of the continuous variables, thrombocytopenia, MELD score, and increased creatinine level were significantly associated with 30-day readmission [17]. In Mousa score, serum albumin < 2.5 g/dL, serum bilirubin > 1.5 mg/dL, serum creatinine > 1.2 mg/dL and INR > 1.4 were significant predictors for 30-day readmission, and so allocated one point for each risky predictive factor. This is in agreement with Bajaj et al. whose model for prediction of readmission in cirrhotic patients using index admission variables showed that lower serum albumin on admission was significantly linked to readmission [19]. In addition, Xu et al. whose risk stratification score to predict readmission in cirrhotic patients found that increased total bilirubin, INR, and serum creatinine, as well as decreased serum albumin comprised significant predictors of readmission in these patients [43].

Mousa score is important in that, it combined unique factors to predict and decrease hospital readmission in cirrhotic patients with SBP based on laboratory and clinical indicators that are easily obtainable at index hospitalization and is easily comparable to next-generation scores. Due to the staggering cost of liver disease in the United States exceeding $2 billion annually in direct healthcare [44], application of the Mousa scoring system for cirrhotic patients with SBP may decrease their hospital readmission thereby lessening the economic burden on medical facilities.

However, this study has several limitations. The relatively small number of cases included in this study is one of our limitations, as well the absence of a validation group. Therefore, it is advised that additional studies including a large number of cases as well as a validated group be conducted.

## Conclusion

The 30-day readmission rate of SBP was noted to be 25.6%. The Mousa readmission risk score is a simple easily applicable score that highlights the need for targeted interventions, to decrease the rates of readmission for patients with SBP. Therefore, using Mousa readmission score may aid in recognizing those at higher risk of readmission.

## Supplementary Information

**Additional file 1: Supplementry figure1**. Box plots to determine thresholds that is reasonable in mousa score.

**Additional file 2: Supplementry table 1**. Risk prediction models for hospital readmission among cirrhotic patients.

## Data Availability

All data generated or analysed during this study are included in this published article.
