# Supplementary figures and images for: A risk score to predict 30-day hospital readmission rate in cirrhotic patients with spontaneous bacterial peritonitis
Source: Eur J Med Res. 2023 May 12;28:168. doi: 10.1186/s40001-023-01126-2 (PMC10176908; doi:10.1186/s40001-023-01126-2)

Figure : Box plots to determine thresholds that is reasonable in mousa score.


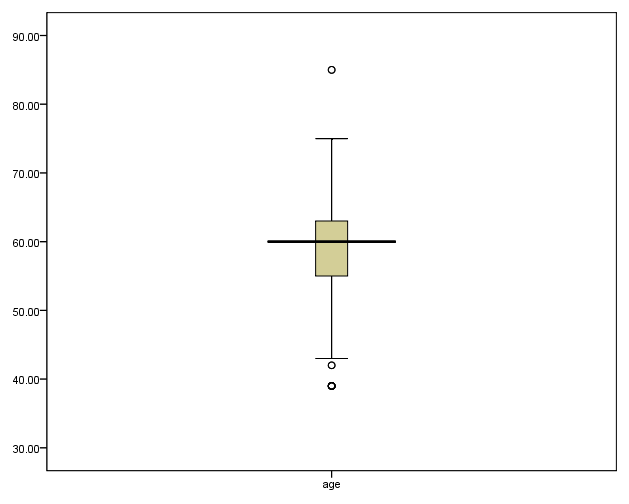


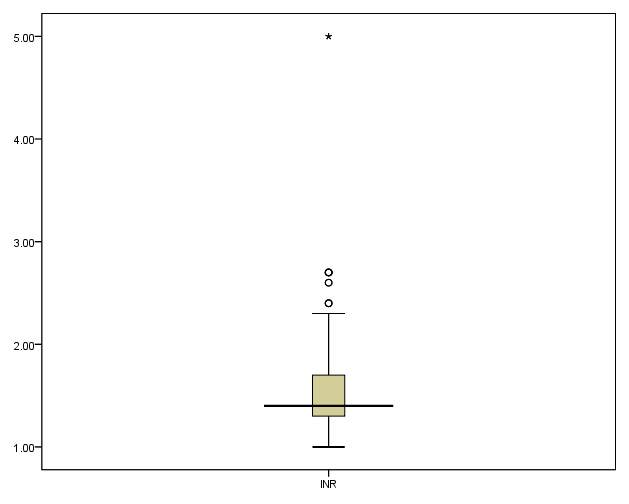

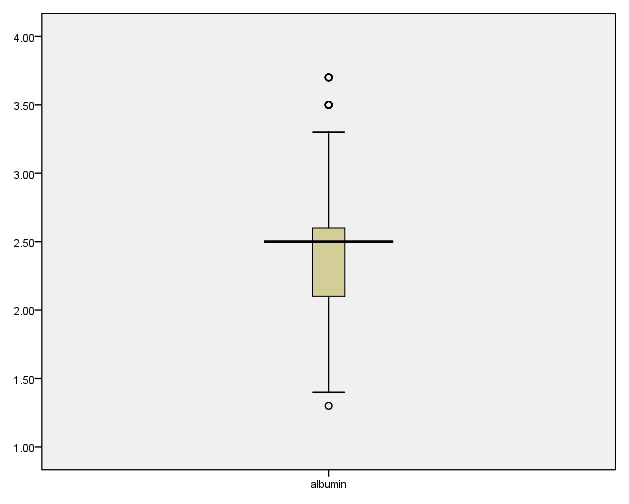


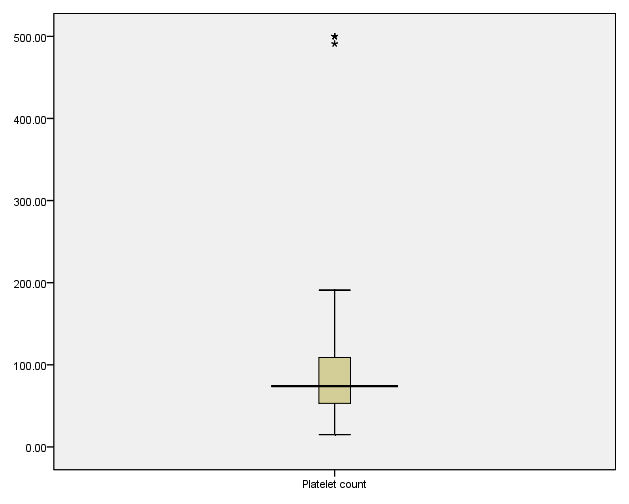


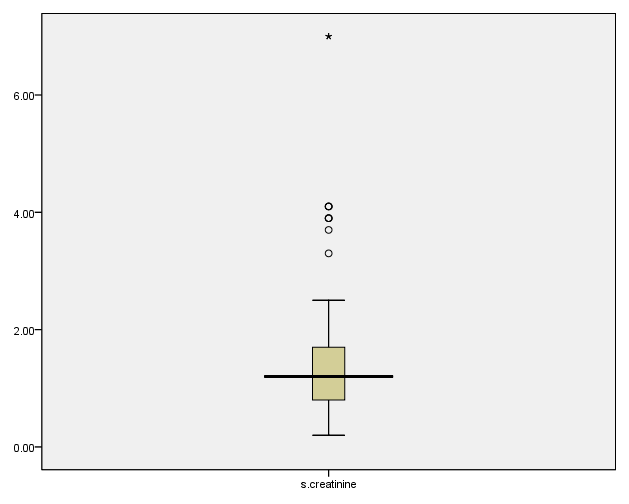


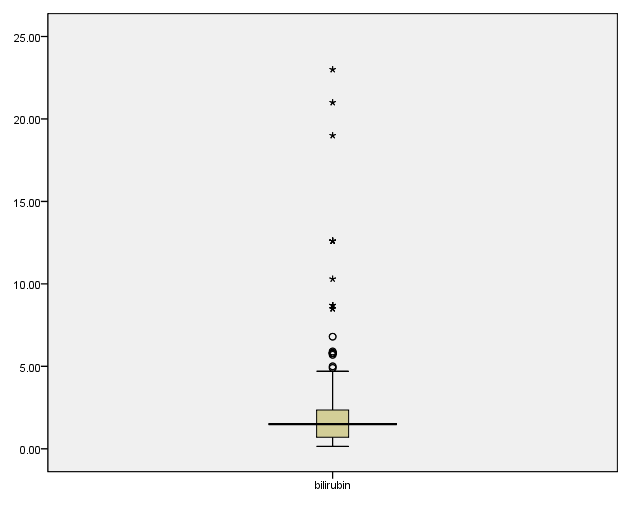

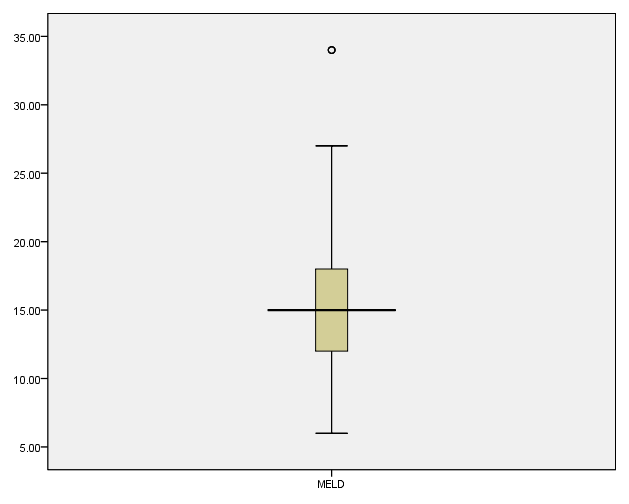

Supplement: Supplementary file 1 — Additional file 1: Supplementry figure1. Box plots to determine thresholds that is reasonable in mousa score. [file 40001_2023_1126_MOESM1_ESM.docx]
