# Supplementary material for: A risk score to predict 30-day hospital readmission rate in cirrhotic patients with spontaneous bacterial peritonitis
Source: Eur J Med Res. 2023 May 12;28:168. doi: 10.1186/s40001-023-01126-2 (PMC10176908; doi:10.1186/s40001-023-01126-2)
Supplement: Supplementary file 2 — Additional file 2: Supplementry table 1. Risk prediction models for hospital readmission among cirrhotic patients. [file 40001_2023_1126_MOESM2_ESM.docx]

| **Model** | **Study** | |  | **No. of** |  | **Candidate Variable** | | | | | | | | | | | | **Discrim-** | **Calib-** |
| --- | --- | --- | --- | --- | --- | --- | --- | --- | --- | --- | --- | --- | --- | --- | --- | --- | --- | --- | --- |
|  | **Type &** | |  | **Candidate** |  |  |  |  |  | **Domains** | | | | | | | | **ination** | **ration** |
|  | **No. of** | |  | **Variables** |  |  |  |  |  |  |  |  |  |  |  |  |  |  |  |
|  | **Subjects** | |  |  |  |  |  |  |  |  |  |  |  |  |  |  |  |  |  |
|  | **D** | **V** |  |  | **D** | **X** | **MP** | | | **I** | **R** | **C** | **L** | **Y** | **U** | **T** | **S** |  |  |
| Berman (2011)^33^ | 447 | 836^¥^ | ~ 27 | |  |  |  |  |  |  |  |  |  |  |  |  |  | C=0.57^¥^ | n/a |
| Bajaj (2016)^35^ | 1343 |  | ~ 25 | |  |  |  |  |  |  |  |  |  |  |  |  |  | C=0.64 | n/a |
| Morales (2017)^13^ | 112 |  | ~ 34 | |  |  |  |  |  |  |  |  |  |  |  |  |  | C=0.76 | HL ns |
| Singal (2013)^34^ | 629 | 209^β^ | ~ 30 | |  |  |  |  |  |  |  |  |  |  |  |  |  | C=0.66 | HL ns |
| Tapper (2015)^37^ | 489 | 245^β^ | ~ 22 | |  |  |  |  |  |  |  |  |  |  |  |  |  | AUC=0.69 | n/a |
| Volk (2012)^36^ | 402 |  | ~ 22 | |  |  |  |  |  |  |  |  |  |  |  |  |  | C=0.65 | n/a |
| Koola (2020)^22^ | 67,749 |  |  | 208 |  |  |  |  |  |  |  |  |  |  |  |  |  |  |  |

Study type = validation (V) and development (D). when a study evaluated performance in a dispersed validation cohort, the performance is stated in the validation cohort. The number of candidate variables had to be concluded *based on* the written methods and the cohort summary table as this number was not explicitly reported. Model discrimination reported as either C-statistic or area under the curve (AUC), which can be considered equivalent. Risk variable domains coding: D: demographics; X: medical/surgical Hx; M: meds; P: inpatient procedures; I: physical impairment; R: risk scores; C: cirrhosis-related complications; L: laboratories; Y: psychosocial; U: healthcare utilization; T: transplant status; S: discharge disposition

^a^Validation performed in separate study (Singal) [18]; β: validation cohorts were made by random train–test split of the original cohort; HL: Hosmer–Lemeshow test for goodness of fit
